# Supplementary material for: High-dose vitamin D3 to improve outcomes in the convalescent phase of complicated severe acute malnutrition in Pakistan: a double-blind randomised controlled trial (ViDiSAM)
Source: Nat Commun. 2025 Mar 15;16:2554. doi: 10.1038/s41467-025-57803-9 (PMC11910567; doi:10.1038/s41467-025-57803-9)
Supplement: Supplementary file 1 — Supplementary Information [file 41467_2025_57803_MOESM1_ESM.pdf]

High-dose vitamin D_3 to improve outcomes in the convalescent phase of complicated severe acute malnutrition in Pakistan: a double-blind randomised controlled trial (ViDiSAM)

Supplementary Information

Contents

Supplementary Methods 2

Randomisation procedure 2

Table S1. Reasons for ineligibility to participate (n=306 participants screened) 3

Table S2. Doses of study preparation received, by allocation (n=259) 4

Table S3. Sub-group analysis: major efficacy outcomes at 2-month follow-up by baseline weight-for-height or -length z-score 4

Table S4. Sub-group analysis: major efficacy outcomes at 2-month follow-up by sex 5

Table S5. Sub-group analysis: major efficacy outcomes at 2-month follow-up by baseline vitamin D status. 6

Table S6. Baseline participant characteristics overall vs. those contributing data to sub-group analysis by baseline vitamin D status 7

Table S7. Serious adverse events by allocation: line listing 9

Statistical Analysis Plan 10

Administrative Information 10

Background and trial design 10

Outcome measures 11

Sample size and randomisation 12

Analysis methods 12

Data summaries 13

References 13

Supplementary Methods

Randomisation procedure

Eligible participants were individually randomised to intervention vs. control arms with a one-to-one allocation ratio and stratification by hospital of recruitment as follows: prior to the start of recruitment, the trial statistician (NF) prepared one hospital randomisation list and two separate participant randomisation lists (one for each site). The hospital randomisation list comprised one pair of letters per participating hospital (Ganga Ram Hospital: A/B, THQ Hospital: Y/Z). One letter within each pair was randomly assigned to the active arm of the trial, and the other was randomly assigned to the placebo arm, using a computer-generated random sequence. The resulting hospital randomisation list was sent to GT Pharma Ltd, Lahore, Pakistan (the manufacturer of the investigational medicinal product [IMP]), with a copy retained by the trial statistician. Neither participants nor trial staff had access to this list. GT Pharma then used this hospital randomisation list to label a total of 600 glass ampoules (300 each containing 5 mg vitamin D_3 in 1 ml ethyl oleate, and 300 each containing 1 ml ethyl oleate without vitamin D_3). The label on each ampoule clearly displayed one of the letters A/B/Y/Z (the randomisation codes indicating whether the ampoule contained active or placebo IMP). Labelled ampoules were then packed into boxes according to the letter code displayed on their label, with each box labelled with the letter corresponding to the contents of the ampoules it contained. Two participant randomisation lists were then generated – one for each hospital site. Each of the participant randomisation lists comprised 999 4-digit numbers consisting of a 1-digit hospital identifier (1 for the Ganga Ram Hospital list, 3 for THQ Hospital), followed by a 3-digit participant identifier from 001 to 999 (e.g. 1-001, 1-002…; 3-001, 3-002 …). These 4-digit numeric codes were then randomly assigned to one or other of the one-letter randomisation codes in use for the corresponding hospital using a computer-generated random sequence employing a blocking structure. When eligibility of a new participant was confirmed (i.e. when the baseline serum albumin-adjusted calcium result was found to be ≤2.65 mmol/L), the participant randomisation list for the relevant site was used to assign IMP to each participant, based on the numeric component of their ID number.

Table S1. Reasons for ineligibility to participate (n=306 participants screened)

**Reason for ineligibility**	**No. of children**[1]
Consent not given	28
Aged < 6 or >59 months	5
Family moved / planned to move out of the study area within 6 months of enrolment	29
SAM without complications or MAM (moderate acute malnutrition)	187
Ingested a dose of vitamin D>200,000 IU (5 mg) in the 3 months prior to enrolment	17
Known diagnosis of primary hyperparathyroidism or sarcoidosis (i.e., conditions pre-disposing to vitamin D hypersensitivity)	0
Known neurodevelopmental disorder (e.g., cerebral palsy)	23
HIV infection	5
Taking anti-tuberculosis treatment	7
Unable to assess child's developmental status at baseline using the Malawi Developmental Assessment Tool	0
Clinical signs of rickets	12
Baseline corrected serum calcium concentration >2.65 mmol/L	7

1, total in this column adds to n=320 because 7 children fulfilled two ineligibility criteria simultaneously

Table S2. Doses of study preparation received, by allocation (n=259)

Total number of doses received	Proportion receiving no. of doses, placebo arm	Proportion receiving no. of doses, vitamin D arm
**0**	1/131 (0.8%)	0/128 (0.0%)
**1**	2/131 (1.5%)	1/128 (0.8%)
**2**	128/131 (97.7%)	127/128 (99.2%)

Table S3. Sub-group analysis: major efficacy outcomes at 2-month follow-up by baseline weight-for-height or -length z-score.

Within-stratum P values are from generalised mixed models, testing for effect of allocation to vitamin D vs placebo with adjustment for baseline value and study site. P values for interaction are from generalised mixed models, testing for effect of allocation to vitamin D vs placebo with adjustment for baseline value and study site, with additional inclusion of an interaction term between the sub-group and allocation to vitamin D vs. placebo. Statistical tests were 2-sided, with no adjustment for multiple comparisons.

Outcome	Baseline weight-for-height or -length z-score	Placebo, mean value (s.d.) [n]	Vitamin D, mean value (s.d.) [n]	Adjusted mean difference (95% CI) *	P value	P for interaction (95% CI)
**Anthropometric/Clinical Outcomes**						
Weight-for-height or -length z-score	≥-3.0	-1.78 (0.96), [31]	-1.86 (0.81), [31]	-0.12 (-0.45, 0.21)	0.48	0.015
	<-3.0	-2.84 (1.01), [95]	-2.83 (1.08), [89]	0.05 (-0.19, 0.29)	0.67	
Lean mass index	≥-3.0	13.03 (2.16), [31]	13.05 (2.24), [31]	0.65 (-0.20, 1.51)	0.14	0.47
	<-3.0	12.50 (2.10), [95]	12.54 (2.41), [89]	-0.04 (-0.62, 0.54)	0.90	
Mean mid-upper arm circumference, cm	≥-3.0	11.69 (0.85), [31]	11.88 (0.78), [31]	0.04 (-0.28, 0.35)	0.82	0.68
	<-3.0	11.51 (0.91), [95]	11.54 (0.92), [89]	0.07 (-0.16, 0.31)	0.53	
Weight-for-age z-score	≥-3.0	-2.99 (1.23), [31]	-2.96 (0.99), [31]	-0.22 (-0.52, 0.08)	0.14	0.14
	<-3.0	-3.67 (1.03), [95]	-3.65 (1.12), [89]	0.08 (-0.13, 0.28)	0.45	
**Neurodevelopmental Outcomes**						
Overall MDAT score	≥-3.0	60.48 (21.56), [31]	60.71 (12.78), [31]	-1.05 (-3.20, 1.10)	0.34	0.43
	<-3.0	62.03 (21.54), [95]	59.37 (23.21), [89]	-0.21 (-1.75, 1.34)	0.79	

*Adjusted for site of recruitment and baseline value. Abbreviations: MDAT: Malawi Developmental Assessment Tool, S.d.: Standard deviation, CI: Confidence interval.

Table S4. Sub-group analysis: major efficacy outcomes at 2-month follow-up by sex. Within-stratum P values are from generalised mixed models, testing for effect of allocation to vitamin D vs placebo with adjustment for baseline value and study site. P values for interaction are from generalised mixed models, testing for effect of allocation to vitamin D vs placebo with adjustment for baseline value and study site, with additional inclusion of an interaction term between the sub-group and allocation to vitamin D vs. placebo. Statistical tests were 2-sided, with no adjustment for multiple comparisons.

**Outcome**	**Sex**	**Placebo, mean value (s.d.) [n]**	**Vitamin D, mean value (s.d.) [n]**	**Adjusted mean difference (95% CI) ***	**P value**	**P for interaction (95% CI)**
**Anthropometric/Clinical Outcomes**						
Weight-for-height/length z-score	Female	-2.43 (1.09), [72]	-2.34 (1.09), [71]	0.03 (-0.23, 0.28)	0.83	0.77
	Male	-2.79 (1.08), [56]	-2.90 (1.05), [52]	-0.06 (-0.40, 0.28)	0.72	
Lean mass index	Female	12.39 (2.08), [72]	12.26 (2.02), [71]	-0.07 (-0.67, 0.52)	0.81	0.22
	Male	12.89 (2.13), [56]	13.19 (2.67), [52]	0.27 (-0.51, 1.05)	0.50	
Mean mid-upper arm circumference, cm	Female	11.50 (0.93), [72]	11.65 (0.77), [71]	0.13 (-0.10, 0.37)	0.26	0.84
	Male	11.67 (0.88), [56]	11.63 (1.04), [52]	-0.02 (-0.33, 0.30)	0.92	
Weight-for-age z-score	Female	-3.35 (1.12), [72]	-3.24 (1.09), [71]	0.03 (-0.18, 0.24)	0.81	0.93
	Male	-3.69 (1.08), [56]	-3.79 (1.08), [52]	-0.02 (-0.31, 0.26)	0.87	
**Neurodevelopmental Outcomes**						
Overall MDAT score	Female	60.43 (19.66), [72]	62.62 (20.36), [71]	0.21 (-1.54, 1.96)	0.82	0.22
	Male	64.77 (25.39), [56]	58.04 (23.74), [52]	-1.18 (-2.95, 0.58)	0.19	

*Adjusted for site of recruitment and baseline value. Abbreviations: MDAT: Malawi Developmental Assessment Tool, S.d.: Standard deviation, CI: Confidence interval.

Table S5. Sub-group analysis: major efficacy outcomes at 2-month follow-up by baseline vitamin D status.

Within-stratum P values are from generalised mixed models, testing for effect of allocation to vitamin D vs placebo with adjustment for baseline value and study site. P values for interaction are from generalised mixed models, testing for effect of allocation to vitamin D vs placebo with adjustment for baseline value and study site, with additional inclusion of an interaction term between the sub-group and allocation to vitamin D vs. placebo. Statistical tests were 2-sided, with no adjustment for multiple comparisons.

Outcome	Baseline 25(OH)D concentration, nmol/L	Placebo, mean value (s.d.), [n]	Vitamin D, mean value (s.d.), [n]	Adjusted mean difference (95% CI)[1]	P for interaction (95% CI)
**Anthropometric/Clinical Outcomes**					
Weight-for-height/length z-score	<50	-2.68 (1.09), [36]	-2.50 (1.08), [31]	0.11 (-0.32, 0.53)	0.96
	≥50	-3.30 (1.14), [16]	-2.63 (1.05), [23]	0.52 (0.02, 1.01)	
Lean mass index, Ω^{-1}	<50	12.88 (2.02), [36]	13.15 (2.87), [31]	0.36 (-0.64, 1.37)	0.23
	≥50	13.26 (3.10), [16]	12.90 (2.25), [23]	-0.42 (-1.73, 0.89)	
Mean mid-upper arm circumference, cm	<50	11.64 (0.82), [36]	11.70 (0.84), [31]	0.08 (-0.26, 0.41)	0.72
	≥50	11.08 (1.34), [16]	11.74 (0.98), [23]	0.47 (-0.08, 1.02)	
Weight-for-age z-score	<50	-3.62 (1.06), [36]	-3.43 (1.10), [31]	0.09 (-0.24, 0.43)	0.44
	≥50	-3.90 (1.26), [16]	-3.61 (1.25), [23]	0.16 (-0.25, 0.57)	
**Neurodevelopmental Outcomes**					
Overall MDAT score	<50	59.14 (21.29), [36]	59.55 (17.91), [31]	0.45 (-2.10, 3.04)	0.38
	≥50	55.38 (20.55), [16]	59.30 (25.19), [23]	0.21 (-3.32, 3.75)	

1, Adjusted for site of recruitment and baseline value. Abbreviations: MDAT: Malawi Developmental Assessment Tool, S.d.: Standard deviation, CI: Confidence interval.

Table S6. Baseline participant characteristics overall vs. those contributing data to sub-group analysis by baseline vitamin D status

		**Overall** (n=259)	**Participants included in subgroup analysis by baseline serum 25(OH)D concentration (n=106)**
**Sociodemographic**			
Mean age, months (s.d.) [range]		14.5 (8.2) [6.0-49.7]	14.0 (7.0) [6.0 to 39.9]
Sex, n (%)	Female	147 (56.8%)	60 (56.6%)
	Male	112 (43.2%)	46 (43.4%)
Ethnic origin, n (%)	Kashmiri	1 (0.4%)	-
	Muhajir	5 (1.9%)	-
	Pashtoon	10 (3.9%)	4 (3.8%)
	Punjabi	211 (81.5%)	93 (87.7%)
	Saraiki	2 (0.8%)	-
	Other	30 (11.6%)	9 (8.5%)
Study site, n (%)	Sir Ganga Ram Hospital	129 (49.8%)	59 (55.7%)
	THQ Hospital	130 (50.2%)	47 (44.3%)
Number of younger siblings at home, n (%)	0	237 (91.9%)	97 (92.4%)
	1	18 (7.0%)	7 (6.7%)
	2 or more	3 (1.2%)	1 (1.0%)
Number of older siblings at home, n (%)	0	42 (16.2%)	14 (13.2%)
	1	74 (28.6%)	38 (35.8%)
	2	57 (22.0%)	22 (20.8%)
	3	37 (14.3%)	9 (8.5%)
	4	31 (12.0%)	14 (13.2%)
	5 or more	18 (6.9%)	9 (8.5%)
Mother's education, n (%)	No formal schooling	92 (35.5%)	44 (41.5%)
	Primary education only	91 (35.1%)	30 (28.3%)
	Secondary education and above	76 (29.3%)	32 (30.2%)
Father's education, n (%)	No formal schooling	96 (37.1%)	46 (43.4%)
	Primary education only	85 (32.8%)	35 (33.0%)
	Secondary education and above	78 (30.1%)	25 (23.6%)
Mean monthly household income, PKR (s.d.)		22575 (14110)	20264 (7576)
Type of residence	Cottage	1 (0.4%)	-
	Flat/apartment	227 (87.6%)	92 (86.8%)

	Terraced house	22 (8.5%)	12 (11.3%)
	Other	9 (3.5%)	2 (1.9%)
Vaccination status, n (%)	Up-to-date	178 (68.7%)	68 (64.2%)
	Not up-to-date	81 (31.3%)	38 (35.8%)
**Early life characteristics**			
Gestational age, n(%)	Term	233 (90.0%)	95 (89.6%)
	Pre-term	26 (10.0%)	11 (10.4%)
Child's birthweight, n (%)	Very low birth weight (<1.5 kg)	2 (0.8%)	-
	Low birth weight (1.5 to < 2.5 kg)	108 (41.7%)	37 (34.9%)
	Normal (≥2.5 kg < 4.0 kg)	146 (56.4%)	67 (63.2%)
	Too heavy (≥ 4.0 kg)	3 (1.2%)	2 (1.9%)
Breastfeeding, n (%)	Still breastfeeding	92 (35.5%)	31 (29.2%)
	Breastfed for 0-5 months then	115 (44.4%)	51 (48.1%)
	Breastfed for 6-12 months then	22 (8.5%)	13 (12.3%)
	Breastfed >12 months then stopped	30 (11.6%)	11 (10.4%)
Age at weaning, months, n (%)	Still exclusively breastfeeding	3 (1.2%)	1 (0.9%)
	<4 months	8 (3.1%)	4 (3.8%)
	4-5 months	67 (25.9%)	27 (25.5%)
	6-8 months	151 (58.3%)	60 (56.6%)
	9-11 months	21 (8.1%)	8 (7.5%)
	12-23 months	9 (3.5%)	6 (5.7%)
**Anthropometry at baseline**			
Mean Weight-for-height or -length z-score		-3.5 (0.9)	-3.6 (0.9)
Mean mid-upper arm circumference, cm (s.d.)		10.5 (0.9)	10.5 (0.9)
Bilateral pitting oedema at hospital admission, n (%)	Present	0 (0%)	0 (0%)
	Absent	259 (100.0%)	106 (100.0%)
Mean weight-for-age z-score (s.d.)		-4.2 (1.0)	-4.2 (1.0)
Mean head circumference z-score (s.d.)		-2.8 (1.2)	-3.9 (0.9)
Lean mass index, Ω^{-1} (s.d.)		11.9 (2.0)	11.9 (2.0)
Complication present at hospital admission, n (%)	Acute lower respiratory infection	51 (19.7%)	14 (13.2%)
	Acute upper respiratory infection	19 (7.3%)	7 (6.6%)
	Anorexia	8 (3.1%)	3 (2.8%)
	Gastroenteritis / Diarrhoea	104 (40.2%)	56 (52.8%)
	Hyperpyrexia	6 (2.3%)	2 (1.9%)
	Hypoglycaemia	1 (0.4%)	-
	Severe anaemia	16 (6.2%)	3 (2.8%)
	Severe dehydration	44 (17.0%)	16 (15.1%)
	Other	10 (3.9%)	5 (4.7%)
**Neurodevelopmental status at baseline**			
Mean overall MDAT score (all domains) (s.d.)		50.8 (21.0)	48.6 (19.9)
Mean Gross motor score (s.d.)		13.6 (5.3)	13.0 (5.3)
Mean Fine motor score (s.d.)		13.9 (5.8)	13.2 (5.4)
Mean Language score (s.d.)		10.4 (5.5)	9.8 (4.9)
Mean Social score (s.d.)		12.9 (5.4)	12.6 (5.3)
**Blood biochemistry**			
Mean total serum 25(OH)D, nmol/L (s.d.)		54.4 (47.3)	54.1 (47.3)
Total serum 25(OH)D category	<25 nmol/L, n (%)	25 (23.4%)	24 (22.6%)
	≥ 25 nmol/L & < 50 nmol/L, n (%)	43 (40.2%)	43 (40.6%)
	≥ 50 nmol/L & < 75 nmol/L, n (%)	19 (17.8%)	19 (17.9%)
	≥75 nmol/L, n (%)	20 (18.7%)	20 (18.9%)

Mean total serum 24R,25(OH)$_2$D, nmol/L		3.1 (4.7)	3.1 (4.7)
Mean total 25(OH)D: total 24R,25(OH)$_2$D ratio		26.1 (10.8)	26.1 (10.8)
Mean serum albumin, g/L (s.d.)		37.7 (5.5)	37.4 (6.1)
Mean serum C-reactive protein, mg/L (s.d.)		5.1 (10.3)	5.2 (10.4)
Mean serum albumin-adjusted calcium, mmol/L (s.d.)		2.38 (0.18)	2.40 (0.20)
Mean serum total alkaline phosphatase, IU/L		278.5 (264.9)	275.4 (265.0)
Mean serum PTH, pmol/L (s.d.)		3.3 (5.7)	3.4 (5.9)
Mean serum ferritin, µg/L (s.d.)		53.8 (83.5)	54.6 (84.1)
Mean serum hepcidin, ng/ml (s.d.)		20.2 (39.1)	20.6 (39.4)
**Urine biochemistry**			
Mean urinary calcium creatinine molar ratio		1.81 (5.75)	1.62 (4.63)
Mean urinary osmolality, mOsm/kg (s.d.)		641.03 (290.42)	593.1 (293.4)
Proportion with urinary calcium:creatinine		71/259 (27.4%)	32/106 (30.2%)
Mean haemoglobin, g/dL (s.d.)		9.0 (1.9)	9.2 (2.0)
Mean corpuscular volume, fL (s.d.)		69.8 (12.9)	70.0 (13.2)
Mean corpuscular haemoglobin		21.3 (4.9)	21.4 (5.1)
Mean total WBC, x10^9/L (s.d.)		12.5 (5.7)	12.8 (6.2)
Mean neutrophil count, x10^9/L (s.d.)		3.9 (2.7)	3.8 (2.2)
Mean lymphocyte count, x10^9/L (s.d.)		6.9 (3.2)	6.9 (3.2)
Mean monocyte count, x10^9/L (s.d.)		1.0 (0.7)	1.1 (0.8)
Mean platelet count, x10^9/L (s.d.)		455 (192)	439 (163)
**Faecal inflammatory markers**			
Mean log-transformed faecal myeloperoxidase, ng/ml (s.d.)		7.5 (1.4)	7.6 (1.5)
Mean log-transformed faecal neopterin, nmol/L (s.d.)		6.9 (1.3)	6.8 (1.4)
Mean log-transformed faecal alpha-1 antitrypsin, mg/dL (s.d.)		2.4 (1.1)	2.5 (1.0)

Abbreviations: THQ Hospital: Tehsil Headquarter Hospital, PKR: Pakistani Rupee, KHz: Kilohertz, MDAT: Malawi Developmental Assessment Tool, WBC: White blood cell, PTH: Parathyroid hormone, 25(OH)D: 25-hydroxyvitamin D, S.d.: Standard deviation, CI: Confidence interval.

Table S7. Serious adverse events by allocation: line listing

	**Placebo arm**	**Vitamin D arm**
Hospitalisation for bronchopneumonia	2	0
Hospitalisation for croup	1	0
Hospitalisation for sepsis	1	0
Hospitalisation for gastroenteritis	6[1]	3
Hospitalisation for viral myocarditis	1[2]	0
**Total**	**11**	**3**

1, of which one was fatal. 2, fatal event

Statistical Analysis Plan

Administrative Information

Study title: Trial of High-Dose Vitamin D in the Treatment of Complicated Severe Acute Malnutrition (ViDiSAM)

Trial registration number: NCT04270643

Version & Date: Statistical Analysis Plan (SAP) v1.0 dated 06FEB2024, based on trial protocol version 3.0, dated 26NOV2020

Members of the writing committee
David Jolliffe was the main author of the statistical analysis plan, with contributions from Adrian Martineau, Nick Freemantle, Javeria Saleem and Andrew Prendergast.

Timing of SAP revisions in relation to unblinding of data/results
David Jolliffe, Adrian Martineau, Javeria Saleem and Andrew Prendergast were blinded to treatment allocation of participants in this study at time this SAP was written and when signed off.

Remit of SAP
The purpose of this document is to provide details of the statistical analyses and presentation of results to be reported to the funder and within the principal papers of the ViDiSAM trial. Subsequent analyses of a more exploratory nature (including those involving baseline data only) will not be bound by this strategy but will be expected to follow the broad principles laid down in it. Any exploratory, *post hoc* or unplanned analyses will be clearly identified in the respective study analysis report.

Background and trial design

**Study objectives**	1. To determine whether high-dose oral vitamin D supplementation increases mean weight-for-height/length z-score in children receiving standard therapy for complicated severe acute malnutrition (primary outcome)<br>2. To determine whether this intervention also enhances neurodevelopment, accumulation of muscle mass and antimicrobial immune function in study participants (secondary outcomes, efficacy)<br>3. To determine whether this intervention is safe and well-tolerated by study participants (secondary outcomes, safety)<br>4. To determine whether effects of high-dose vitamin D are modified by baseline vitamin D status (sub-group analysis)
**Study design**	Double-blind, parallel-group individually randomised placebo-controlled clinical trial.
**Setting**	In-patient and Out-patient Health Care Centres in Lahore, Pakistan
**Participants**	Main inclusion criteria<br>• Consent of parent / guardian

	• Age 6-59 months at enrolment<br>• Diagnosis of complicated severe acute malnutrition at the point of hospital admission<br>• Medical team managing the child has made the decision to discharge the child from inpatient care<br><br>Main exclusion criteria<br><br>• Ingestion of a dose of vitamin D>200,000 IU (5 mg) in the last 3 months<br>• Known diagnosis of primary hyperparathyroidism or sarcoidosis (i.e. conditions pre-disposing to vitamin D hypersensitivity)<br>• Known neurodevelopmental disorder (e.g. cerebral palsy)<br>• HIV infection<br>• Taking anti-tuberculosis treatment<br>• Inability to assess child's developmental status at baseline using the Malawi Developmental Assessment Tool<br>• Clinical signs of rickets<br>• Baseline corrected serum calcium concentration >2.65 mmol/L
**Interventions**	Intervention arm: two oral doses of 200,000 IU (5 mg) vitamin D_3 in 1 ml ethyl oleate, administered at point of hospital discharge and 2 weeks thereafter.<br><br>Control arm: two oral doses of placebo IMP (identical to Intervention IMP except that no Vitamin D contained)$_3$, administered at point of hospital discharge and 2 weeks thereafter.
**Primary outcome measure(s)**	Mean weight-for-height or -length z-score at 2-month follow-up timepoint

Outcome measures

Primary outcome

Mean weight-for-height or -length *z-score* at 2-month follow-up, adjusted for baseline, calculated using World Health Organization (WHO) child growth standards

Secondary outcomes

<u>Efficacy</u>

1. Mean weight-for-height or -length *z-score* at 6-month follow-up, adjusted for baseline
2. Other anthropometric outcomes at 2- and 6-month follow-up, adjusted for baseline (weight-for-age, height/length-for-age, head circumference-for-age, mid-upper arm circumference [MUAC])
3. Mean neurodevelopmental scores (Malawi Developmental Assessment Tool) at 2- and 6-month follow-up, adjusted for baseline, both for total scores and for scores relating to separate domains (gross motor, fine motor, language and social)
4. Lean mass index at 2- and 6-month follow-up, adjusted for baseline (Bodystat 1500 bioimpedance analyser)

5. Proportion of participants experiencing relapse of SAM (complicated or uncomplicated) during 6-month follow-up
6. Antimicrobial immune function at 2- and 6-month follow-up, adjusted for baseline (concentrations of inflammatory mediators in supernatants of whole blood stimulated with LPS, zymosan and heat-killed *S. typhi*)
7. Serum concentrations of albumin, C-reactive protein, 25(OH)D (the accepted biomarker of vitamin D status), total alkaline phosphatase, parathyroid hormone, ferritin, hepcidin, TLR4 ligands, type II Interferon activity and other inflammatory mediators at 2- and 6-month follow-up, adjusted for baseline
8.Urinary ratios of calcium: creatinine and urine osmolality at 2- and 6-month follow-up, adjusted for baseline
9. Full blood count indices (mean haemoglobin concentration, mean corpuscular volume, mean corpuscular haemoglobin concentration, mean total and differential white blood cell counts) at 2- and 6-month follow-up, adjusted for baseline
10. Faecal concentrations of inflammatory markers (myeloperoxidase, neopterin and alpha-1 antitrypsin) at 2- and 6-month follow-up, adjusted for baseline
11. Composition of the faecal microbiome at 2- and 6-month follow-up, adjusted for baseline

<u>Safety</u>
1. Mortality
2. Incidence of serious adverse events
3. Incidence of hypercalcaemia
4. Incidence of any other adverse event attributed to study medication

Sub-group analyses
Influence of allocation on efficacy end-points detailed above will be separately evaluated in:
a) Participants with vs. without vitamin D deficiency at baseline,
b) males vs. females.
c) Participants with baseline WFHZ <-3 v.s WHFZ ≥3

Sample size and randomisation

Sample size calculation:
Assuming a mean 2-month weight-for-height/length z-score of -2.0 in the control arm, with standard deviation 1.25 and 20% loss to follow-up, a total of 250 participants (125 per arm) will provide 80% power to detect an inter-arm difference in weight-for-height/length z-score (primary outcome) of 0.50 (i.e. -1.5 vs. -2.0 in intervention vs control arms, respectively) with alpha =0.05. This effect size is conservative compared to our previous finding of +1.07 Z-score gain with this regimen in n=185 children with uncomplicated SAM [1].

Randomisation procedure:
Randomisation was carried out by Nick Freemantle on a 1:1 ratio to the two treatment arms. Treatment was allocated in randomly permuted blocks of 10 (5 to control, 5 to intervention within a block) within participating hospitals. The blocks reflect the ordering of within-hospital participant IDs (so for hospital with code 01, the first block allocates treatments to participants with IDs 01-001 to 01-010 inclusive, the second block to participants 01-011 to 01-020 etc.).

Analysis methods

Analysis Principles

All statistical analyses will be performed on an intention-to-treat basis, and conducted two-sided. P values <0.05 will be interpreted as significant. Outcome variables' distribution will be assessed and where non-normal, data will be log-transformed prior to analysis. All follow-up efficacy outcomes will be adjusted for site of recruitment and their baseline value. There is no hierarchical structure for secondary and safety outcomes listed in the appendix and there will be no adjustment for multiplicity for these outcomes when they are analysed.

Primary outcome
The effect of allocation on the primary outcome of mean weight-for-height or -length z-score at 2-month follow-up will be analysed using generalised mixed models, including baseline and endpoint values for each subject, linked with random intercept terms. The models will test for effect of allocation to vitamin D vs placebo with adjustment for baseline value and study site (which was a stratification factor).

Secondary outcomes
The effect of allocation on secondary efficacy outcomes will be analysed as follows: Continuous outcomes (such as mean anthropometric z-scores, neurodevelopmental scores, lean mass index) will be analysed using analogous models to the primary outcome. For analysis of MDAT data, raw developmental scores may be transformed to scaled scores prior to analysis, using an item response theory (IRT) statistical model. Dichotomous efficacy outcomes (such as the proportion of participants experiencing a given outcome at least once) will be analysed using non-linear mixed models with a logit link and binomial / mixed error, again analogous to the primary analysis. The effect of allocation on safety outcomes (proportions of participants experiencing a given adverse event) will be investigated in participants who took one or more doses of IMP and will be described narratively. Immunological parameters in whole blood supernatants and serum/plasma will be analysed according to the principles above (i.e. with adjustment for baseline value and study site); where multiple inflammatory mediators are analysed.

Interim Analysis

Open and closed reports were reviewed by the DMC on 4th of April 2023; they recommended continuation of the study.

Pre-specified Subgroup Analysis
Heterogeneity of treatment effect will be examined among sub-groups defined by baseline vitamin D status, sex, and SAM diagnostic criteria (children with baseline MUAC <115 mm and baseline WHZ ≥-3.0 vs. children with baseline WHZ <-3.0 irrespective of baseline MUAC). For these analyses, the primary analysis will be repeated with additional inclusion of an interaction term between the sub-group and allocation to vitamin D vs. placebo, and a P value for interaction between allocation and sub-group will be presented.

Data summaries

Data will be presented in tabular and graphical forms.

References

1. Saleem, J., et al., *High-dose vitamin D3 in the treatment of severe acute malnutrition: a multicenter double-blind randomized controlled trial.* Am J Clin Nutr, 2018. **107**(5): p. 725-733.
