# Supplementary material for: High-dose vitamin D3 to improve outcomes in the convalescent phase of complicated severe acute malnutrition in Pakistan: a double-blind randomised controlled trial (ViDiSAM)
Source: Nat Commun. 2025 Mar 15;16:2554. doi: 10.1038/s41467-025-57803-9 (PMC11910567; doi:10.1038/s41467-025-57803-9)
Supplement: Supplementary file 2 — Reporting Summary [file 41467_2025_57803_MOESM2_ESM.pdf]

Corresponding author(s): Adrian Martineau
Last updated by author(s): Aug 29, 2024

Reporting Summary

Nature Portfolio wishes to improve the reproducibility of the work that we publish. This form provides structure for consistency and transparency in reporting. For further information on Nature Portfolio policies, see our Editorial Policies and the Editorial Policy Checklist.

Statistics

For all statistical analyses, confirm that the following items are present in the figure legend, table legend, main text, or Methods section.

n/a	Confirmed	
☐	☒	The exact sample size (*n*) for each experimental group/condition, given as a discrete number and unit of measurement
☒	☐	A statement on whether measurements were taken from distinct samples or whether the same sample was measured repeatedly
☐	☒	The statistical test(s) used AND whether they are one- or two-sided<br>*Only common tests should be described solely by name; describe more complex techniques in the Methods section.*
☐	☒	A description of all covariates tested
☐	☒	A description of any assumptions or corrections, such as tests of normality and adjustment for multiple comparisons
☐	☒	A full description of the statistical parameters including central tendency (e.g. means) or other basic estimates (e.g. regression coefficient) AND variation (e.g. standard deviation) or associated estimates of uncertainty (e.g. confidence intervals)
☐	☒	For null hypothesis testing, the test statistic (e.g. *F*, *t*, *r*) with confidence intervals, effect sizes, degrees of freedom and *P* value noted<br>*Give P values as exact values whenever suitable.*
☒	☐	For Bayesian analysis, information on the choice of priors and Markov chain Monte Carlo settings
☒	☐	For hierarchical and complex designs, identification of the appropriate level for tests and full reporting of outcomes
☐	☒	Estimates of effect sizes (e.g. Cohen's *d*, Pearson's *r*), indicating how they were calculated

Our web collection on statistics for biologists contains articles on many of the points above.

Software and code

Policy information about availability of computer code

Data collection	REDCap v14
Data analysis	Stata software (Version 17.0; StataCorp, College Station, Texas, United States) and SAS (Version 9.4; SAS Institute, Cary NC)

For manuscripts utilizing custom algorithms or software that are central to the research but not yet described in published literature, software must be made available to editors and reviewers. We strongly encourage code deposition in a community repository (e.g. GitHub). See the Nature Portfolio guidelines for submitting code & software for further information.

Data

Policy information about availability of data

All manuscripts must include a data availability statement. This statement should provide the following information, where applicable:

- Accession codes, unique identifiers, or web links for publicly available datasets
- A description of any restrictions on data availability
- For clinical datasets or third party data, please ensure that the statement adheres to our policy

The data and code generated in this study have been deposited in the codeocean database under accession code 2673880 (https://codeocean.com/capsule/2673880/tree). Source data are provided with this paper.

Research involving human participants, their data, or biological material

Policy information about studies with human participants or human data. See also policy information about sex, gender (identity/presentation), and sexual orientation and race, ethnicity and racism.

Reporting on sex and gender	The trial recruited males and females. Sub-group analyses were conducted to test for effect modification by sex.
Reporting on race, ethnicity, or other socially relevant groupings	Ethnic origin of participants, overall and by allocation, is presented in Table 1 (Kashmiri, Muhajir, Pashtoon, Punjabi, Saraiki, Other)
Population characteristics	Population characteristics are presented in Table 1. Inclusion criteria were consent of the parent or guardian; participant age of 6-59 months at enrolment; a diagnosis of complicated SAM at the point of hospital admission, as defined by the World Health Organisation (WHO) (29); and planned discharge from in-patient care. Exclusion criteria were ingestion of a dose of vitamin D >200,000 IU (5 mg) in the last 3 months; known diagnosis of primary hyperparathyroidism or sarcoidosis (i.e. conditions pre-disposing to vitamin D hypersensitivity); known neurodevelopmental disorder (e.g. cerebral palsy); HIV infection; taking anti-tuberculosis treatment; inability to assess a child's developmental status at baseline using the Malawi Developmental Assessment Tool (MDAT) (30); clinical signs of rickets; child's family planning to move out of the study area within 6 months of enrolment; and baseline albumin-adjusted serum calcium concentration >2.65 mmol/L.
Recruitment	Participants were recruited from the Sir Ganga Ram Hospital (SGRH) and the Tehsil Headquarter (THQ) Hospital, in Lahore, Pakistan. Parents or legal guardians were invited to provide written informed consent for their child to participate in the trial.
Ethics oversight	The trial was ethically approved by the National Bioethics Committee (NBC) of Pakistan (ref. 4-87/NBC-516/20/477) and the Liverpool School of Tropical Medicine Research Ethics Committee, UK (ref. 20-028).

Note that full information on the approval of the study protocol must also be provided in the manuscript.

Field-specific reporting

Please select the one below that is the best fit for your research. If you are not sure, read the appropriate sections before making your selection.

☒ Life sciences ☐ Behavioural & social sciences ☐ Ecological, evolutionary & environmental sciences

For a reference copy of the document with all sections, see nature.com/documents/nr-reporting-summary-flat.pdf

Life sciences study design

All studies must disclose on these points even when the disclosure is negative.

Sample size	Assuming a mean 2-month WHZ of -2.0 in the control arm, with a standard deviation of 1.25 and 20% loss to follow-up, we calculated that a total of 250 participants (125 per arm) would allow us to detect an inter-arm difference in WHZ of 0.50 (-1.5 vs. -2.0 in intervention vs control arms, respectively) with 80% power and 5% type 1 error.
Data exclusions	No data were excluded from the analysis
Replication	Not applicable - no experimental findings in this clinical trial
Randomization	Eligible participants were individually randomised to intervention vs. control arms with a one-to-one allocation ratio and stratification by hospital of recruitment using computer-generated random sequences; a full description of how this was done is presented in Supplementary Material.
Blinding	Treatment allocation was concealed from participants' parents or guardians, their medical care providers, and all trial staff (including senior investigators and those assessing outcomes) so that the double-blind was maintained.

Reporting for specific materials, systems and methods

We require information from authors about some types of materials, experimental systems and methods used in many studies. Here, indicate whether each material, system or method listed is relevant to your study. If you are not sure if a list item applies to your research, read the appropriate section before selecting a response.

Materials & experimental systems

n/a	Involved in the study
☒	☐ Antibodies
☒	☐ Eukaryotic cell lines
☒	☐ Palaeontology and archaeology
☒	☐ Animals and other organisms
☐	☒ Clinical data
☒	☐ Dual use research of concern
☒	☐ Plants

Methods

n/a	Involved in the study
☒	☐ ChIP-seq
☒	☐ Flow cytometry
☒	☐ MRI-based neuroimaging

Clinical data

Policy information about clinical studies

All manuscripts should comply with the ICMJEguidelines for publication of clinical research and a completedCONSORT checklist must be included with all submissions.

Clinical trial registration	ClinicalTrials.gov ref NCT04270643
Study protocol	Full protocol included with submission
Data collection	Data collected at the Sir Ganga Ram Hospital (SGRH) and the Tehsil Headquarter (THQ) Hospital, in Lahore, Pakistan, from December 2021 to February 2023.
Outcomes	The primary outcome was the difference between active and placebo participants in mean weight-for-height or -length z-score (WHZ) at 2 months after administration of the first dose of IMP, calculated using WHO child growth standards (32) and accounting for baseline values. Secondary efficacy outcomes were inter-arm differences in mean WHZ at 6-month follow-up; mean weight-for-age z-score, height- or length-for-age z-score, head circumference-for-age z-score, mid-upper arm circumference and lean mass index (calculated as 1000/impedance at 50 kHz) at 2- and 6-month follow-up; mean MDAT scores (overall and gross motor, fine motor, language and social scores separately) at 2- and 6-month follow-up; serum concentrations of albumin, C-reactive protein, total 25-hydroxyvitamin D (25[OH]D, the accepted biomarker of vitamin D status), total 24R,25-dihydroxyvitamin D (24R,25[OH]2D, the major catabolite of 25[OH]D), ratios of serum concentrations of total 25(OH)D to total 24R,25[OH]2D (indicating the degree of interconversion of these metabolites), total alkaline phosphatase, parathyroid hormone, ferritin and hepcidin at 2- and 6-month follow-up; mean urinary molar ratios of calcium:creatinine and mean urine osmolality at 2- and 6-month follow-up; full blood count indices (mean haemoglobin concentration, mean corpuscular volume, mean corpuscular haemoglobin concentration, mean total and differential white blood cell counts) at 2- and 6-month follow-up; and median faecal concentrations of inflammatory markers (myeloperoxidase, neopterin and alpha-1 antitrypsin) at 2- and 6-month follow-up. Safety outcomes were mortality, incidence of serious adverse events, the proportion of participants experiencing relapse of SAM (complicated or uncomplicated) over 6-month follow-up, and incidence of hypercalcaemia (defined as serum albumin-adjusted calcium >2.65 mmol/L), hypercalciuria (defined as urinary molar calcium: creatinine ratio >1.00), hypervitaminosis D (defined as serum 25[OH]D concentration >220 nmol/L) or any other adverse event attributed to IMP.

Plants

Seed stocks	N/A
Novel plant genotypes	N/A
Authentication	N/A
